# Attenuated alpha–gamma coupling in emotional dual pathways with right‐Amygdala predicting ineffective antidepressant response

**DOI:** 10.1111/cns.13787

**Published:** 2021-12-24

**Authors:** Zhongpeng Dai, Cong Pei, Siqi Zhang, Shui Tian, Zhilu Chen, Hongliang Zhou, Qing Lu, Zhijian Yao

**Affiliations:** ^1^ Key Laboratory of Child Development and Learning Science School of Biological Sciences & Medical Engineering Child Development and Learning Science Research Center for Learning Science Ministry of Education Southeast University Nanjing China; ^2^ Department of Psychiatry The Affiliated Brain Hospital of Nanjing Medical University Nanjing China; ^3^ Nanjing Brain Hospital Medical School of Nanjing University Nanjing China

**Keywords:** alpha–gamma coupling, amygdala, antidepressant response, dual pathways, magnetoencephalography, negative stimuli

## Abstract

**Aims:**

The diversity of treatment outcomes for major depressive disorder (MDD) remains uncertain in neuropathology. The current study aimed at exploring electrophysiological biomarkers associated with treatment response.

**Methods:**

The present study recruited 130 subjects including 100 MDD patients and 30 healthy controls. All subjects participated in a sad expression recognition task while their magnetoencephalography data were recorded. Patients who had a reduction of at least 50% in disorder severity at endpoint (>2 weeks) were considered as responders. Within‐frequency power and phase‐amplitude coupling were measured for the brain regions involved in the emotional visual information processing pathways.

**Results:**

The significant alpha–gamma decoupling from the right thalamus to the right amygdala in unconscious processing and from right orbital frontal cortices to the right amygdala in conscious processing was found in non‐responders relative to responders and healthy controls. These kinds of dysregulation could also predict the potential treatment response.

**Conclusion:**

The attenuated alpha–gamma coupling in dual pathways indicated increased sensitivity to the negative emotional information and reduced moderated effect of the amygdala, which might cause insensitivity to antidepressant treatment and could be regarded as potential neural mechanisms for treatment response prediction.

## Graphical abstract

The attenuated alpha‐gamma interactions in both awareness and unawareness paths associated with antidepressant response. Specifically, these aberrant frequency interactions mainly involved brain regions in emotional dual model, like right amygdala, right thalamus, right orbital frontal cortices. The disrupted oscillatory activity could be utilized to predict treatment response at baseline.

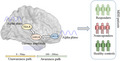

## 1 INTRODUCTION

Major depressive disorder (MDD) is a prevalent psychiatric disease which seriously affects the quality of life and results in a tremendous socioeconomic burden.^1^ Although a range of antidepressants have been widely used in clinical practice, only less than one‐half of patients obtained remission after their first course of treatment.^2^ As treatment selection by trial and error caused increasing patient disability and suicide risk, achieving individualized treatment response prediction at baseline is an urgent and vital task. With the limited predictive capacity of demographic and clinical variables,^3^ neuroimaging studies have revealed some pretreatment biomarkers in relation to antidepressant response.^4^, ^5^

A promising perspective to find biomarkers of antidepressant response was analyzing neural activity of patients under emotional stimuli. Previous studies have suggested a dysfunction of top–down regulation to negative stimuli between the prefrontal cortex and the amygdala in MDD, exhibiting negative emotional biases compared with healthy people.^6^, ^7^ Sufficient evidences supported that successful pharmacotherapy could modulate emotional information processing and ameliorated the negative biases of MDD patients.^8^, ^9^ Moreover, emotional modulation of antidepressants was much earlier than changes occurring in clinical symptoms. Based on this finding, a clinical study revealed that there was a significant correlation between the increased accuracy in the facial recognition task over the first 2 weeks of antidepressants and the clinical improvement after 6 weeks.^10^ Furthermore, change in emotional information processing over the first week of citalopram was suggested to be a predictor of antidepressant response at day 56.^11^

Behavioral studies that further explored the process of negative emotional biases in MDD patients suggested that these negative biases were mediated by the rapid implicit pathway involving the amygdala below the level of conscious awareness.^12^ A dual‐routed model was proposed to explain that the amygdala might play a crucial role in emotional processing through two parallel pathways: a subcortical pathway (thalamus‐amygdala) and a cortical pathway (frontal cortex‐amygdala).^13^ The subcortical pathway through which the thalamus directly regulated the activity of the amygdala was crude but rapid (<50 ms), and it was considered to be an automatic activation for avoiding danger before further information processing.^14^ More information was then processed by the cortical pathway involving the orbitofrontal and prefrontal cortex after the activation of the visual cortex.^15^ Based on the dual‐routed model, functional magnetic resonance imaging (fMRI) studies revealed that baseline activation of the pregenual anterior cingulate cortex and amygdala under explicit emotional face stimuli might constitute biomarkers of antidepressant response.^16^, ^17^ However, due to the limited temporal resolution, finer information in the time‐frequency domain involving the parallel pathways requires further exploration.

Utilizing electroencephalography (EEG) with higher temporal resolution, studies have investigated oscillatory neural activity in MDD patients in different frequency bands.^4^ The frontal alpha activity was revealed to be associated with the processing of sad expressions, and even influenced neuropathology and social cognition function. And the activation in the low‐frequency band was enhanced for angry facial expressions compared to happy facial expressions.^18^ Furthermore, elevated high‐frequency neural activity in the gamma‐band was observed during the processing of emotional stimuli.^19^ Intracranial recordings have revealed that the amygdala and the orbitofrontal cortex show enhanced gamma‐band activity in response to negative facial expressions.^20^ Disrupted visual gamma modulations were suggested to induce abnormal facial emotion processing in MDD.^21^ Besides, between‐frequency characteristics between gamma power to the phase of low‐frequency oscillations have identified to be associated with modulated mechanisms of emotional and cognitive functions in different neurology disorders^22^, ^23^, ^24^; although, a lot of neurophysiological evidence implied that the abnormal frequency‐specific characteristics might be associated with emotional processing dysfunctions in depression.^25^ Analyses which explored neuronal spectral oscillatory modulated differences in the elaborate parallel pathways of emotional processing were still rare to date.

Based on the previous literatures, we hypothesized that interactions between regions involved in the dual‐routed mode under the negative stimuli may be associated with antidepressant response and deserved to be further probed via within‐frequency or cross‐frequency measurements. To test these hypotheses, magnetoencephalography (MEG) was utilized in the current study as it could capture rapid oscillations in specific neural circuits.^26^ Thus, the relation between antidepressant response and dysregulated frequency specific interactions can be further extrapolated during both the conscious and unconscious pathways under the negative emotional task.

## 2 MATERIALS AND METHODS

### 2.1 Participants

The current analysis recruited a total of 130 participants from the Affiliated Brain Hospital affiliated with Nanjing Medical University between November 2016 and December 2019, including 30 HC and 100 MDD patients. All MDD patients were diagnosed with the Mini‐International Neuropsychiatric Interview (MINI, Chinese version), according to Diagnostic and Statistical Manual of Mental Disorders Fourth Edition, Text Revision (DSM‐IV‐TR).^27^ Depressive symptom severity was assessed with the 17‐item Hamilton Depression Rating Scale (HAM‐D 17), and the severity of anxiety was assessed using the Hamilton Anxiety Rating Scale (HAMA) respectively.^28^ The inclusion criteria for patients were as follows: (1) right‐handed and native Han Chinese; (2) depressive episode with a HAM‐D‐17 total score >17; (3) no comorbidity with other mental disorders, such as schizophrenia, substance abuse, and obsessive compulsive disorders; (4) no systematic psychotherapy or physical therapy in the past six months. Besides, participants were excluded if they had (1) serious medical conditions such as organic brain disorders or severe somatic diseases assessed by past medical history or laboratory analysis; (2) history of alcohol and drug abuse; (3) pregnancy.

All MDD participants received monotherapy with selective serotonin reuptake inhibitors (SSRIs). No systematic psychological intervention such as cognitive behavior therapy was performed. The medication doses were as follows: escitalopram (10–20 mg/day, *n* = 45), sertraline (100–200 mg/day, *n* = 36), fluoxetine (20–60 mg/day, *n* = 19). Considering the dominant treatment effect, the 6‐item Hamilton Depression Rating Scale (HAM‐D‐6) was chosen to assess the treatment response in this study as it is reported to be highly sensitive to the disease variation.^29^ As the first 2 weeks of antidepressant treatment could predict subsequent treatment outcomes, and psychiatrists can decide to continue or change treatment earlier, the 2 weeks is regarded as an endpoint in the experiment process.^30^, ^31^ Thus, patients were initially categorized by the reduction ratios of HAM‐D 6 scores after the first 2 weeks: (1) with reduction ratios <50% constituted the non‐responder group. (2) with a reduction ratio ≥50% were included in the responder group. Thus, the demographic and clinical characteristics of all remaining participants are summarized in Table 1.

**TABLE 1:** Demographic and clinical characteristics of the participants^a^

	Responders (*N* = 32)	Non‐responders (*N* = 33)	Healthy controls (*N* = 29)	*p* Value
Age (years)	31.43 ± 9.03	32.70 ± 9.36	30.62 ± 6.87	0.63^b^
Gender (female/male)	14/18	16/17	13/16	0.92^c^
Education (years)	13.69 ± 2.99	13.06 ± 2.77	14.05 ± 2.78	0.21^b^
Family history of psychiatric illnesses (Yes/No)	7/25	9/24	—	0.64^c^
Current period (Month)	5.79 ± 4.51	6.71 ± 5.96	—	0.51^d^
HAMD‐17	22.32 ± 5.21	24.85 ± 5.43	—	0.19^d^
HAMD‐6	11.20 ± 2.81	12.35 ± 2.10	—	0.14^d^
Escitalopram	17	16	—	—
Sertraline	10	11		
Fluoxetine	5	6		

^a^ Data were presented as the mean (±SD). The scores of HAMD‐17, HAMD‐6, and items of HAMD‐6 are recorded at baseline. The *p* value more than 0.05 indicated no statistical significance between the two groups.

^b^ The *p* value was obtained by univariate ANOVA test.

^c^ The *p* value was obtained by two‐tailed Pearson's chi‐square test.

^d^ The *p* value was obtained by the two‐sample two‐tailed *t* test.

The whole study was approved by the Local Medical Ethics Committee at the Affiliated Brain Hospital of Nanjing Medical University and abided the ethical guidelines of the World Medical Association Declaration of Helsinki. All participants provided written informed consents.

### 2.2 Task stimuli and data acquisition

All participants were instructed to attend a passively viewing task of negative emotional faces. The task included 40 sad expressions, which were selected from the Chinese facial expression video system. Each picture was displayed for 3 s, succeeded by a blank screen with a variable stimulus interval of 0.5 s, 1 s, and 1.5 s.

Magnetoencephalography data were recorded using the Omega 2000, 275 channel CTF MEG system with a 1200 Hz sampling rate. The participants were instructed to lie in the supine position while performing the visual task in a magnetically shielded room. Furthermore, structural T1 data were acquired with a Siemens Verio 3T MRI system using a high‐resolution, T1‐weighted, 3D gradient‐echo pulse sequence. The detailed scanning parameters were as follows: repetition time (TR) = 1900 ms, echo time (TE) = 2.48 ms, flip angle (FA) = 9°, slice thickness = 1 mm, number of slices = 176, acquisition voxel size = 1 × 1 × 1 mm^3^, matrix = 256 × 256, and field of view (FOV) = 250 × 250 mm^2^. Earplugs containing vitamin E were placed in ear canals for offline registration between structural MRI and MEG data.

### 2.3 Data preprocessing and source reconstruction

The MEG data were preprocessed with the Matlab‐based Fieldtrip toolbox.^32^ First, 50‐Hz power line interference was removed with a band‐stop filter, and a band‐pass filter with a 1–100 Hz cut‐off followed. Then, trials and channels containing excessive variance were removed. Note that the number of removed trials was not significantly different between the three groups (non‐responder: 7.3 ± 4.1; responder: 7.6 ± 3.6; HC: 7.5 ± 4.5, *p* = 0.82). Moreover, an independent component analysis (ICA) was further applied to remove eye blink and movement and cardiac and muscle artifacts with visual inspection.

Source level power was estimated with a linearly constrained minimum variance (LCMV) beamformer, which was utilized to project the resulting preprocessed MEG data onto a regular 6‐mm grid source space.^33^ The LCMV beamformer constructed a spatial filter which passes the signals for each time point to a predefined source while minimizing the contributions of other sources. Individual source models were warped to a common Montreal Neurological Institute space. The spatial filters were constructed from the lead fields for each grid and the covariance matrix of signal data. A multivariate symmetric orthogonalization was utilized to weaken the spatial leakage interferences, which could be an effective symmetric approach for removing correlations between ROIs induced by leakage.^34^

### 2.4 Regions of interest definition and source power analysis

**FIGURE 1:**
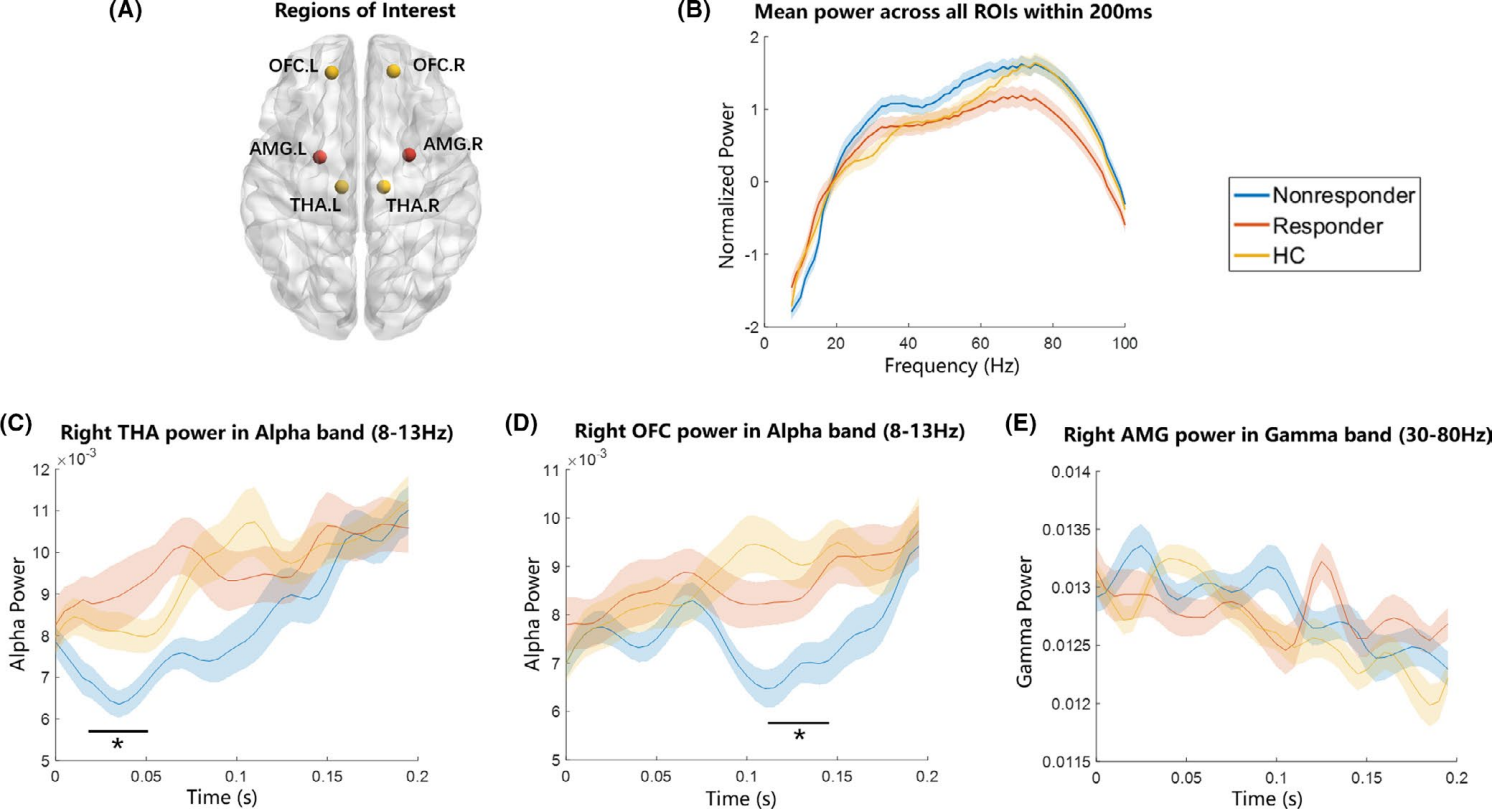
Within‐frequency power across groups. (A) Regions of interest (ROIs): right/left amygdala (AMG), right/left thalamus (THA), and right/left orbital frontal cortices (OFCs) (B) Mean power cross all ROIs within 200 ms. There were no significant differences in power between three groups. (C) Right THA power in the alpha band (8–13 Hz). There existed significant differences between three groups in the 0–50 ms period. (D) Right OFC power in the alpha band (8–13 Hz). There existed significant differences between three groups in 100–150 ms period. (E) Right AMG power in gamma band (30–80 Hz). There was no significant difference in gamma power between three groups. The shaded area around each curve indicated 95% confidence intervals

To quantify the relationship between subcortical and cortical pathways for early emotional processing and treatment response in MDD patients, we selected six regions of interests (ROIs): bilateral amygdala (AMG), bilateral thalamus (THA), and bilateral orbital frontal cortices (OFCs) defined via the Automated Anatomical Labeling (AAL) template atlas (Figure 1A).^35^ Specifically, the source power of each brain region was extracted by averaging the power of all source voxels contained in the corresponding template. By computing the spectral power for each region and then averaging over different groups, the mean power spectra across each region were normalized by dividing the sum of power estimates in the whole frequency domain from 1 to 100 Hz. Then, frequency‐specific powers of dominant frequency components were calculated for each region in each participant using time‐frequency representation (TFR). For the low‐frequency band (1–30 Hz), an adaptive time window of four cycles per frequency multiplied by a Hanning taper was applied, and for a high‐frequency band (30–100 Hz), three orthogonal Slepian tapers with a 50‐ms fixed sliding window was used.^21^

### 2.5 Cross‐frequency coupling estimation

After measuring the local within‐frequency oscillatory activity, cross‐frequency interactions between different frequency bands were also deserved to be further explored in order to investigate the regional modification. Considering the rich neurophysiological implications of alpha and gamma bands in emotional processing, the indirect regulation mechanism between these two frequency bands was then probed in all the ROIs. Alpha–gamma interactions during the unconscious emotional processing stage and the conscious emotional stage were calculated in the period of 0–50 ms and 100–200 ms after stimuli onset, respectively. Cross‐frequency coupling (CFC), which measures how the phase of slower oscillations is coupled to the power of neuronal activity in higher frequency bands, has been measured in brain regions in various conditions. The CFC was quantified by calculating the coherence between the phase of low‐frequency oscillatory activity and the amplitude of high‐frequency oscillation cross‐regions.^36^ Specifically, we performed the analysis using the phase of frequencies from 8 to 13 Hz, at the step of 1 Hz, and the amplitude of envelope for frequencies from 30 to 100 Hz, with the increment of 1 Hz each. Consequently, the CFC matrix for each subject in each region was estimated as two 51 × 18 matrices (phase frequency bins × amplitude frequency bins), respectively, in rapid implicit processing and conscious emotional processing.^22^

### 2.6 Statistical analysis

After ensuring the continuous variables with normal distribution, an independent *F* test was performed in averaged alpha (8–13 Hz) and gamma (30–80 Hz) power between non‐responder, responder, and HC groups in each time bin (within 200 ms after stimulus onset). The resulted *p* values that survived the False Discovery Rate (*FDR*) correction were retained for the post hoc paired comparisons between responder and non‐responder groups via an independent two‐sample *t* test. A non‐parameter cluster‐based permutation test was performed in CFC matrices between non‐responder,^37^ responder, and HC groups in various regional pairs. This procedure forms clusters of neighboring frequency bins and amplitude bins, thus effectively controlling for multiple comparison testing. Group labels were randomly shuffled 1000 times to get the reference cluster distribution. For each iteration, the clusters of *t* values were applied to construct the reference distribution. Cluster statistic values exceeding the 95th percentile of the reference cluster distribution were considered significant (*p* < 0.05). Then, we extracted and averaged amplitude‐phase values in each participant and compared them with the two‐sample *t* test (non‐responder vs. responder, HC vs. responder).

## 3 RESULTS

### 3.1 Demographic and clinical characteristics

Of the initial 130 participants, nine patients received an electroconvulsive therapy due to their illness condition and 10 participants including one HC and nine patients showed excessive head motions or poor image quality. Besides, 17 patients were switched to serotonin–norepinephrine reuptake inhibitor (SNRI) therapy. Of the remaining 65 depressed patients, (1) 33 patients with reduction ratios of HAM‐D‐6 < 50% constituted the non‐responder group. (2) 32 patients with a reduction ratio of HAM‐D‐6 ≥ 50% were included in the responder group. Thus, the demographic and clinical characteristics of all 94 participants were summarized in Table 1. No statistically significant differences were found between the three groups in the demographic and clinical variables.

### 3.2 Decreased alpha oscillatory activity in AMG and THA related to non‐responders

In each group, all the ROIs manifested strong power activation in the gamma band and weak power activation in the alpha band within 200 ms after stimuli onset (Figure 1B). Interestingly, there were no significant differences among three groups in gamma or alpha time‐locked powers within 200 ms after stimuli onset (*p* > 0.05, *FDR* correction). Furthermore, TFR analyses indicated that significant attenuated alpha oscillatory power in the right THA was found in the non‐responder group relative to responder and HC groups from around 20 to 50 ms after stimuli onset (non‐responder vs. responder: *p* < 0.01, survived *FDR* correction, Figure 1C). Meanwhile, significantly decreased alpha band power in the right OFC was found in the non‐responder group than other groups from around 110 to 145 ms after stimuli onset (non‐responder vs. responder: *p* < 0.01, survived *FDR* correction, Figure 1D). These evidence in alpha frequency replicated regions in dual pathways theory. Interestingly, there were no significant differences in gamma power among three groups even in the right AMG which played an essential role in primary emotion regulation (Figure 1E).

### 3.3 Decreased time‐varying alpha–gamma CFC associated with non‐responders

**FIGURE 2:**
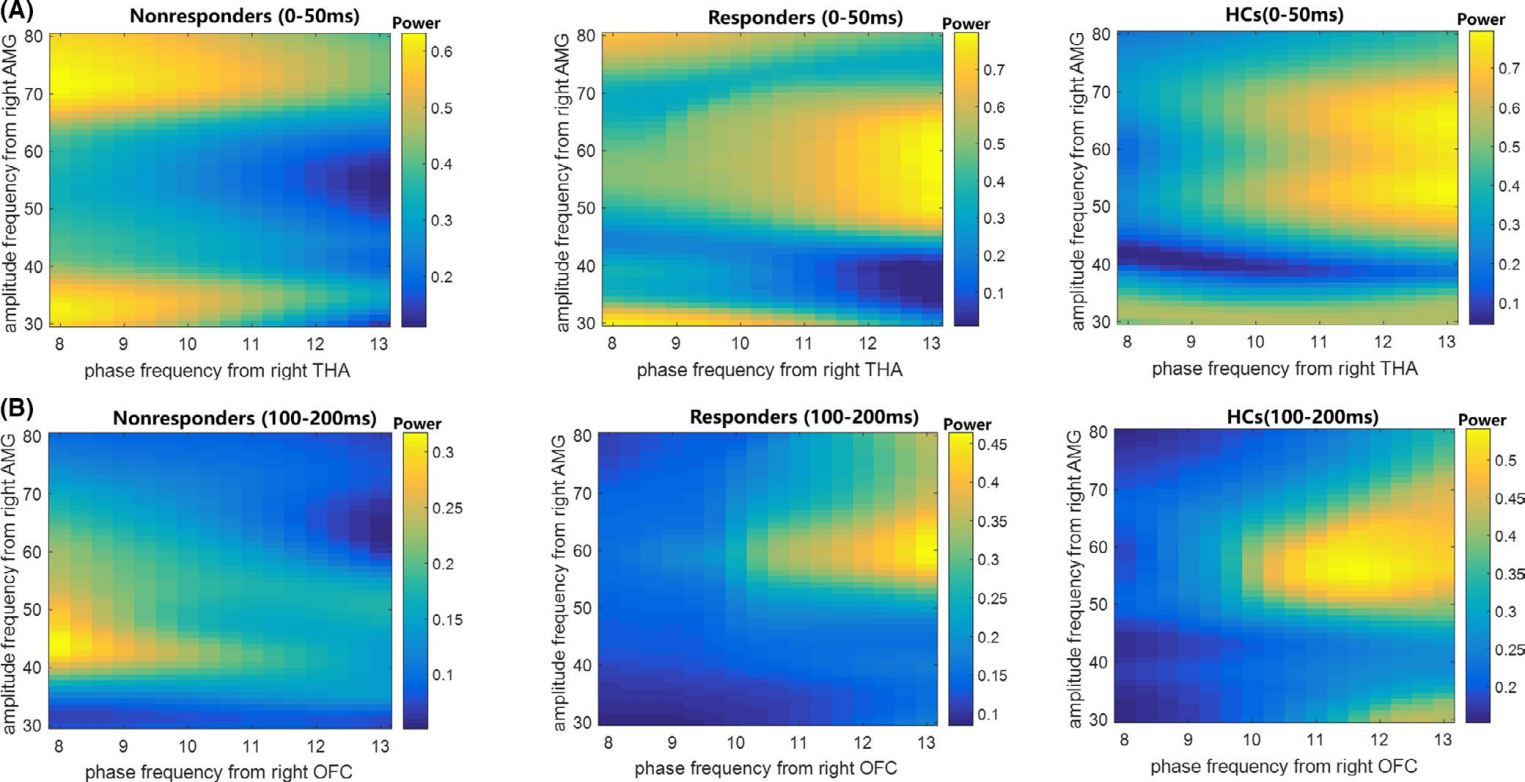
Between‐frequency coupling across groups. (A) Non‐responders manifested attenuated alpha–gamma PAC between the right thalamus (THA) and right amygdala (AMG) in 0–50 ms after stimuli onset relative to responders and HCs. (B) The non‐responders manifested attenuated alpha–gamma PAC between the right OFC and right AMG in 100–200 ms after stimuli onset relative to responders and HCs

Since all regions manifested strong gamma power and weak alpha power, and significantly decreased alpha power was observed in the right THA and right OFC of the non‐responder group, the association between the strong alpha and weak alpha was deserved to be further probed, especially for non‐responder group. To explore whether abnormal interactions such as decoupling between frequencies has disturbed the emotional processing and then affected the treatment response, the strengths of regional cross‐frequency interactions along the rapid implicit path and conscious awareness path were estimated. We calculated CFC matrices between 8–13 Hz alpha phase power and 30–80 Hz gamma amplitude power within two stages (rapid implicit stage: 0–50 ms; conscious awareness stage: 100–200 ms) across all ROIs for each participant, forming the CFC comodulograms. The averaged CFC comodulograms of non‐responders, responders, and HCs in the rapid implicit path are shown in Figure 2A respectively. Meanwhile, averaged CFC comodulograms of these three groups in the conscious awareness path are shown in Figure 2B respectively.

**FIGURE 3:**
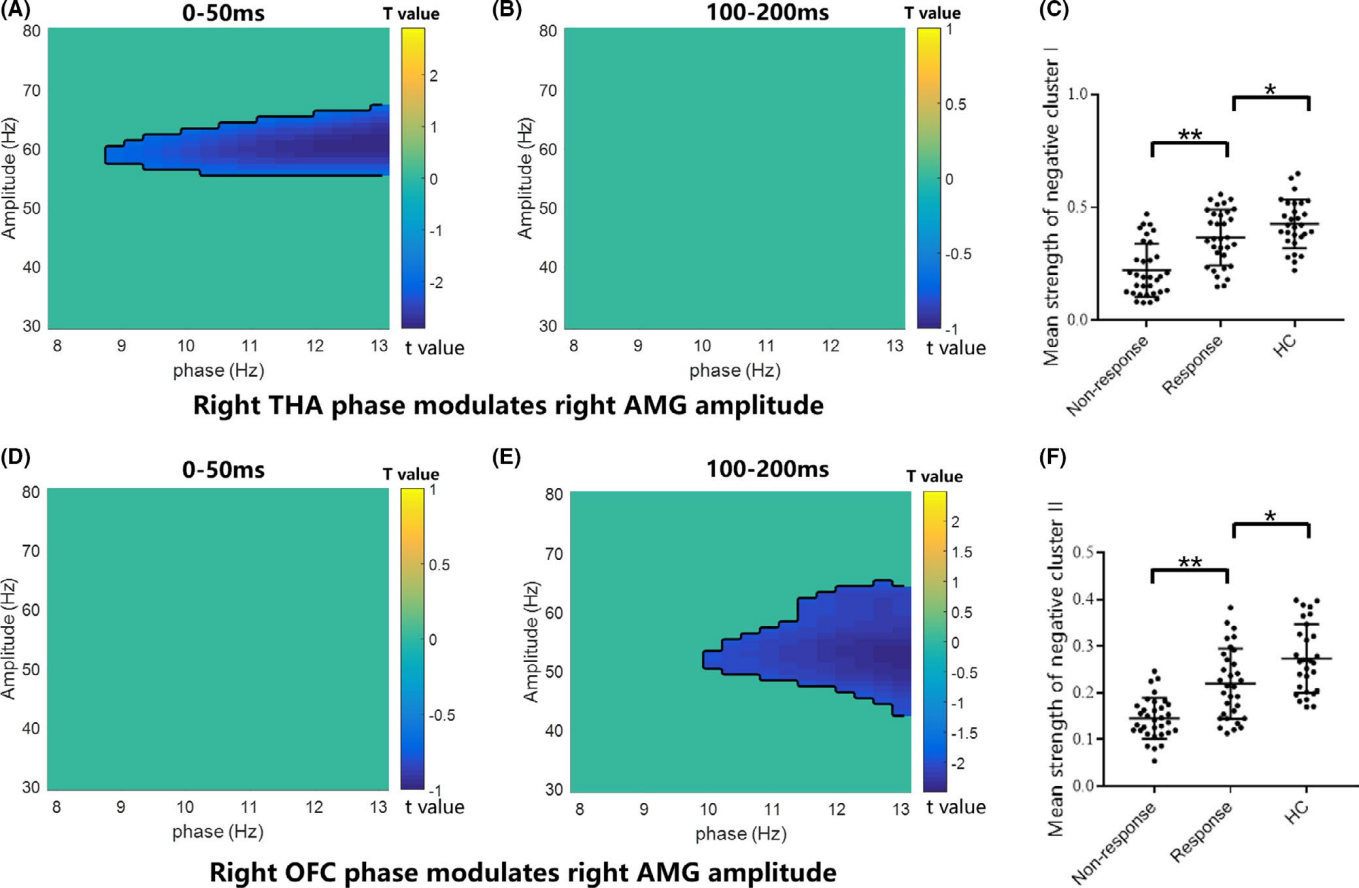
Statistical results of PAC differences between groups. (A‐B) Alpha–gamma PAC between the right THA and right AMG in 0–50 ms was statistically attenuated in non‐responders relative to responders and HCs via the non‐parameter cluster‐based permutation test. (C) Mean strength of negative cluster in (A) between three groups. (D‐E) Alpha–gamma PAC between the right OFC and right AMG in 100–200 ms was statistically attenuated in non‐responders relative to responders and HCs via the non‐parameter cluster‐based permutation test. (F) Mean strength of negative cluster in (E) between three groups. *p < 0.01 **p < 0.0001

Non‐parametric cluster‐based permutation tests were performed to statistically evaluate group differences between HC group, responder group, and non‐responder group. There was a negative cluster of attenuated CFC between THA and AMG in the rapid implicit pathway (*p* < 0.01) (Figure 3A). No significant cluster of CFC between the THA and AMG was detected in the conscious awareness pathway (Figure 3B). Then, we averaged values in the negative cluster and compared them in three groups. As exhibited in Figure 3C, the non‐responder group showed significantly decreased strength relative to the responder group (*p* < 0.001, after *FDR* correction). Similarly, there was a negative cluster of decreased CFC between the OFC and AMG in the automatic awareness pathway (*p* < 0.05) (Figure 3E), and there was no significant cluster for these regions in the rapid implicit pathway (Figure 3D). As shown in Figure 3F, the non‐responder group exhibited significantly decreased cluster strength relative to other two groups (*p* < 0.001, after *FDR* correction). The full unedited Figure 3A‐B, D‐E was exhibited in the Figure S2A‐D.

### 3.4 AMG‐relevant CFC decoupling correlated to reduction ratios of HAM‐D‐6

**FIGURE 4:**
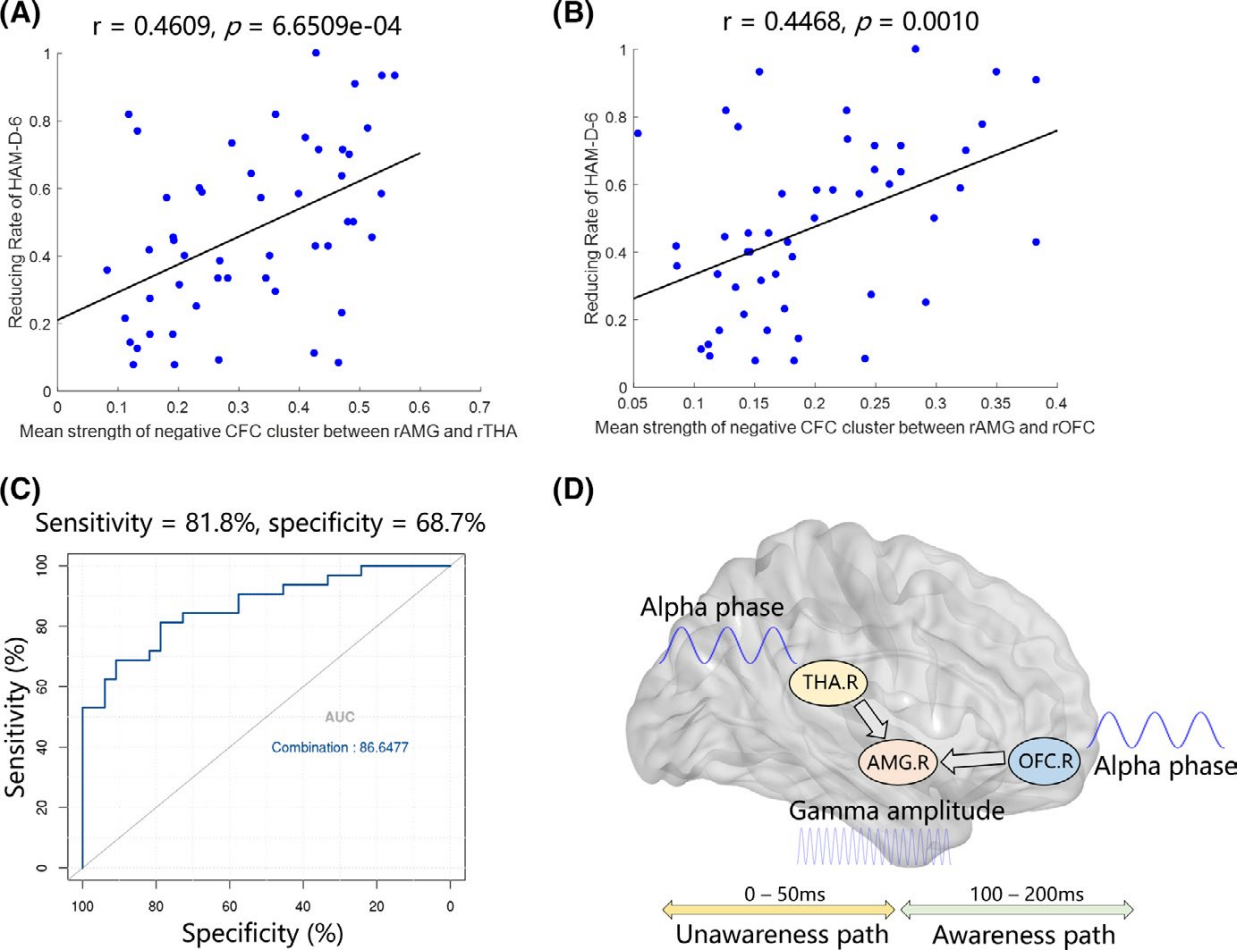
Prediction of antidepressant response with PAC decouplings. (A‐B) Strong correlation between reduction ratios of HAM‐D 6 and PAC decoupling. (C) ROC curve discriminating non‐responders from responders. (D) Schematic of the neural oscillatory relation between the right THA, right OFC, and right AMG in unawareness (0–50 ms) and awareness pathways (100–200 ms)

Reduction ratios of HAM‐D‐6 in all participants were correlated with the mean strength of two negative clusters referred above. Specifically, there were significant positive correlations between reduction ratios and mean strength of negative clusters, which characterized the interaction between the THA and AMG during 0–50 ms (*p* = 6.6509 × 10^−4^, *r* = 0.4609) (Figure 4A) and the interaction between the OFC and AMG during 100–200 ms (*p* = 0.001, *r* = 0.4468) (Figure 4B). These findings indicated that the mean strength of negative clusters might have good discriminative power of antidepressant response at the baseline. Besides, to probe the discriminative power of both interactions, we first performed Receiver Operating Characteristic (ROC) analysis on these two abnormal interactions, respectively, and subsequently combined both interactions. The predictive curve indicated that the combination of the averaged strength of two significant negative clusters have better discriminate non‐responders from responders via the linear regression (Figure 4C, AUC = 0.866, sensitivity = 81.8%, specificity = 68.7%) than the single interaction (Figure S1). These phenomena suggested that decreased AMG‐relevant interactions in alpha–gamma CFC involving the subcortical pathway during the rapid implicit stage and cortical pathway during the conscious emotional processing stage were closely related to antidepressant response in MDD and could predict antidepressant prognosis. The detailed schematic of the dysregulated neural oscillatory representations in dual pathways is exhibited in Figure 4D.

## 4 DISCUSSION

In this study, early perceptual processing of sad expressions based on the dual‐routed model was suggested to be potential for antidepressant response prediction. By analyzing the cross‐frequency coupling between ROIs, we demonstrated that decreased PAC values between rTHA‐rAMG of the subcortical pathway (unconscious rapid implicit pathway) and rOFC‐rAMG of the cortical pathway (conscious awareness pathway) could distinguish non‐responders from responders and HCs (Figure 4D). Besides, the features extracted from PAC matrices were further confirmed to be potential biomarkers of baseline prediction for treatment response.

The dual‐routed model revealed that the activity of AMG was successively modulated by the THA and OFC in the negative emotion processing. By comparing the modulation patterns of the AMG among three groups, we found that the significantly attenuated alpha–gamma interaction with rTHA and rAMG was shown particularly in the unconscious processing stage (0–50 ms), while the significantly decreased alpha–gamma interaction with rOFC and rAMG was shown particularly in the conscious processing stage (100–200 ms) in the non‐responder group. This phenomenon was corresponding to previous studies of emotional processing^38^, ^39^ and enhanced the understanding of antidepressant response prediction through the dual‐routed model. Notably, both findings in AMG‐involving pathways were obtained via cross‐frequency coupling, which might be caused by the initial status of the top–down regulation in many neural functions.^40^ Thus, the dysfunction of cross‐frequency interaction in unconscious processing aroused by inactive rAMG may lead to more serious negative emotional biases and finally result in poor effect of antidepressants. Meanwhile, the dysfunction in the cortical pathway may influence the primary conscious processing of emotional contents, such as emotional arousal and value assessment. As the AMG is the core structure of human emotional function, the rAMG was revealed to be related to autonomic arousal generated by negative emotions and plays a critical role in regulating emotion during the early perceptual processing.^41^ Besides, previous studies showed that the over‐activation of the AMG under sad emotional stimuli was related to the unconscious processing of emotional information, which might lead to negative emotional biases of patients with MDD.^42^ Therefore, current results on antidepressant response prediction further suggested that the over‐activated amygdala at baseline may predict the non‐response of the following medications. The activation of the AMG under negative emotional stimuli was important for emotional dysfunction and antidepressant response in MDD patients. Furthermore, in primary processing of emotional information, the activation of the AMG is regulated by brain regions, such as the THA and OFC. Focusing on the early stage of emotion processing (0–200 ms), we further confirmed this neural modulation based on subcortical and cortical pathways. Compared with the responders, the non‐responders showed a lack of modulation of the rAMG in both pathways. The inactive rAMG, which loses the spectral modulation control to other regions, may be associated with poor antidepressant response in non‐responders.

Another essential issue in this study is the effects of hemisphere asymmetric engagement of aberrant cross‐frequency interaction in emotion processing on the antidepressant response. The results reported that the significant differences of PAC between ROIs only existed in the right hemisphere (0–50 ms rTHA‐rAMG, 100–200 ms rOFC‐rAMG). It should be noticed that there is no evidence of lateralization in the dual‐routed model. For this consideration, such a phenomenon may be explained from the following two aspects. First, according to the valence theory of emotion processing, the left hemisphere is responsible for processing positive emotions while the right hemisphere is responsible for processing negative emotions.^43^ There is over‐expression of sadness in patients with MDD, and this mood‐congruent effect is related to the neural circuit of the right hemisphere.^44^ Therefore, in response to the sad emotional stimuli, the related neural circuits in the right hemisphere may be over‐activated compared with the left hemisphere, thus showing significant differences between the responders and the non‐responders. On the other hand, there is functional lateralization of the bilateral AMG when processing different forms of emotional information. Previous studies have revealed that the left AMG is closely related to emotional information coding, mainly for the processing of the verbal information and detail features which are relatively complicated, while the right AMG is more involved in the rough but rapid processing of the emotional information.^45^ The experimental paradigm in this study was based on emotional facial recognition where pictures presented the facial expressions directly. Therefore, the right AMG may be more involved in this visual task than the left AMG. As a result, the related neural circuits (subcortical and cortical pathways) of the right AMG may also be more effective in predicting the treatment response.

For neural activation in the specific frequency domain, although few studies utilized the cross‐frequency coupling to explore the modulation pattern of brain regions in specific neural circuits, previous studies have found some potential regional predictors of antidepressant response in the specific frequency domain. As neural oscillation in the gamma band plays a key role in integrating cognitive and emotional information, a large number of studies revealed that MDD patients showed reduced activation of the rAMG in the gamma band.^46^ Moreover, the activation in the gamma band was also revealed to be able to predict the 6‐week response of paroxetine.^47^ In this study, no significant difference among the three groups was found by time‐frequency analysis of each brain region. However, based on the activation pattern in the frequency domain, alpha and gamma bands were selected, and significant difference was demonstrated by PAC analysis. These results suggested that the neural pattern which is related to the response of antidepressants may be reflected by the alpha–gamma cross‐frequency interaction between ROIs rather than the gamma‐band activation of a single ROI.

From the clinical perspective, our study found positive correlations between the mean strength of the significant clusters extracted from PAC matrices and reducing rate of HAMD‐6. Previous studies showed that the restoration of interaction between the relevant brain regions in responders was positively correlated with the improvement in symptoms.^48^ Our results of correction analysis further confirmed that features obtained from cross‐frequency modulation in subcortical and cortical pathways at baseline were also correlated with the symptom improvement after 2‐week antidepressant treatment in patients with MDD. Notably, the alpha–gamma interactions of the responder group were closer to the HC group relative to the non‐responder group, which suggested that responders might be more likely to recover from the decoupling state, while the non‐responders might be more difficult to recover to a normal level after 2 weeks' medical treatment. Furthermore, the ROC analysis indicated that the attenuated alpha–gamma PAC values between regions involved in the dual‐routed model at baseline could serve as a predictor of the response of the antidepressants.

Several issues should also be taken into consideration. First, the current study mainly focused on the fine dual‐routed emotional pathways rather than visual areas. However, recent studies have shown that there was the association between aberrant gamma oscillations in the visual cortex and emerging psychosis.^46^ Depressed patients showed abnormal dynamics in visual networks during the similar emotional task^49^; thus, future study could further explore the relation between impaired visual areas and emotional dysfunction. Second, as the current study aimed at exploring dual‐routed pathways in the early emotional processing stage, analyses were performed in limited brain regions rather than large‐scale brain networks, which might also be associated with antidepressant response via previous literatures.^50^

Besides, there were still several limitations in this study. First, although MEG data are relatively rare due to its expensive cost for collecting, the small sample size still limited the reliability of our findings in neural mechanism. Second, although a group of healthy subjects was applied as the control, the current study was still a cross‐sectional study and lacked longitudinal neural data to further verify. Third, the current study only explored the impaired neural mechanism under the pure sad expressions. Considering the diversity of mechanisms underlying the processing of emotions, future paradigms should take more kinds of emotional faces into consideration, such as happiness, fear, and anxiety to explore the more elaborate impairment of neural circuits.

In summary, the current study found that both the attenuated alpha–gamma interactions in the implicit unconscious processing stage (0–50 ms) and conscious awareness processing stage were associated with antidepressant response. Specifically, these aberrant frequency interactions were mainly caused by brain regions in the dual‐routed model, which involved the rAMG in both cortical and subcortical neural pathways. Besides, this kind of disrupted oscillatory activity could be utilized to predict treatment response at baseline.

## CONFLICT OF INTEREST

The authors have no conflict of interest to declare.

## AUTHOR CONTRIBUTIONS

Conceptualization, Q.L. and Z.Y.; Methodology, Z.D. and C.P.; Formal Analysis, Z.D., S.Z., and C.P.; Investigation, Z.D., S.Z., C.P., and Z.C; Writing – Original Draft, Z.D.; Writing – Reviewing & Editing, Z.D, Q.L, S.Z, S.T, and H.Z.; Visualization, Z.D.; Supervision, Q.L. and Z.Y.; Funding Acquisition, Q.L. and Z.Y.

## Supporting information

Figure S1

Figure S2

## Data Availability

The data and codes that support the findings of this study are available on request from the corresponding author. The data are not publicly available due to privacy or ethical restrictions.
