# Supplementary figures and images for: Attenuated alpha–gamma coupling in emotional dual pathways with right‐Amygdala predicting ineffective antidepressant response
Source: CNS Neurosci Ther. 2021 Dec 24;28(3):401–10. doi: 10.1111/cns.13787 (PMC8841302; doi:10.1111/cns.13787)

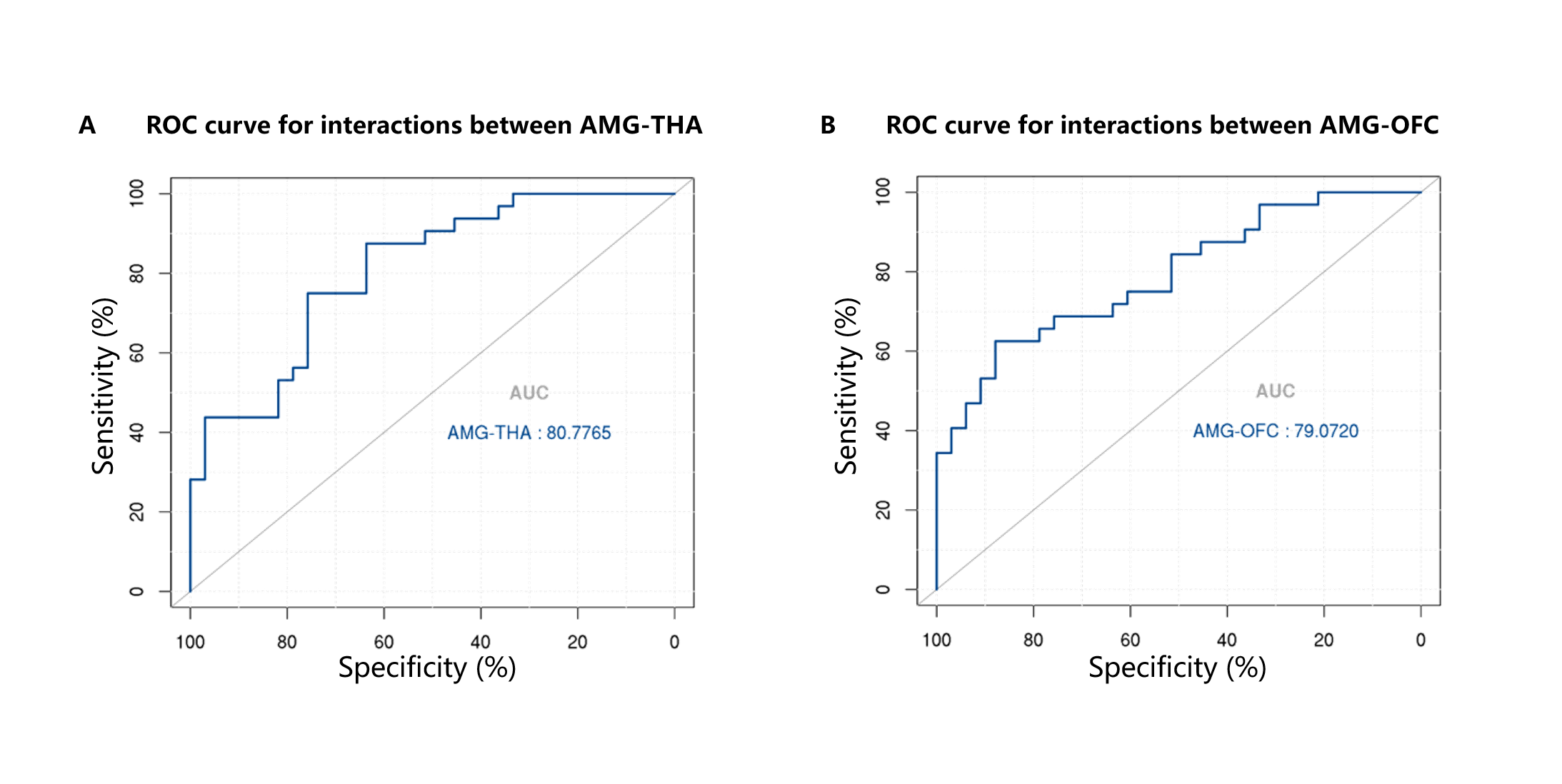

Supplement: Supplementary file 1 — Figure S1 [file CNS-28-401-s002.tif]

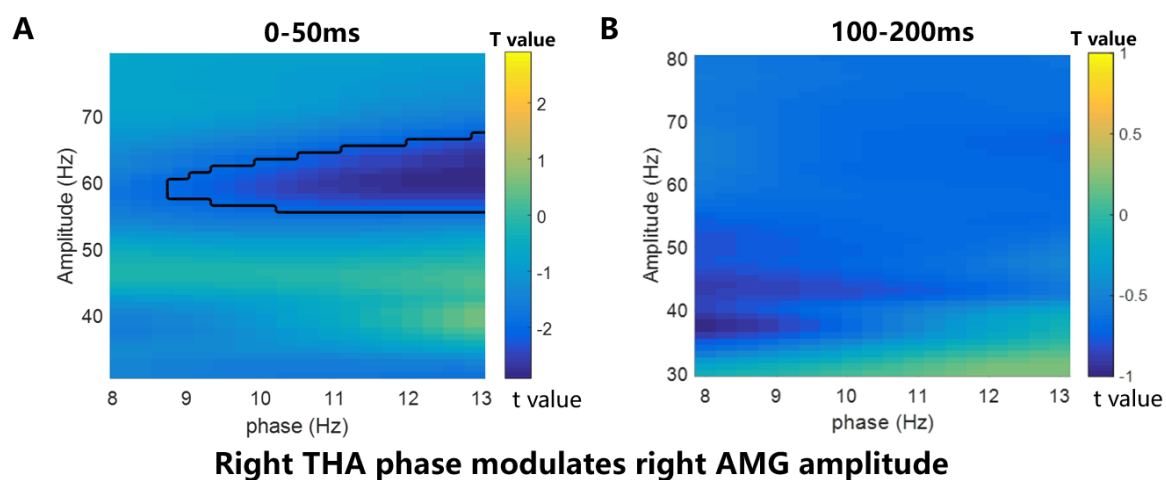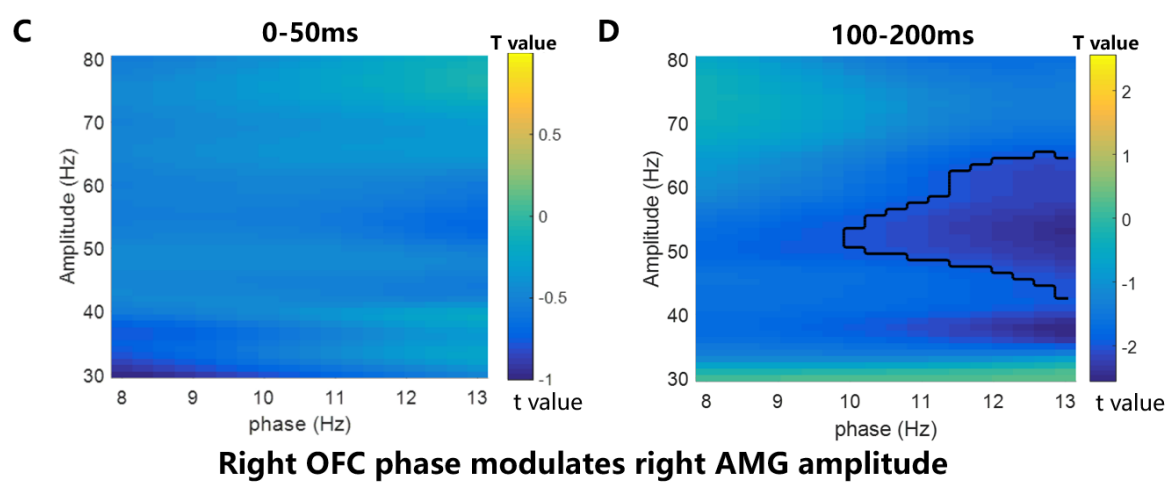

Supplement: Supplementary file 2 — Figure S2 [file CNS-28-401-s001.pdf]
